# Temporal change in Syndecan-1 as a therapeutic target and a biomarker for the severity classification of COVID-19

**DOI:** 10.1186/s12959-021-00308-4

**Published:** 2021-08-16

**Authors:** Fumihiro Ogawa, Yasufumi Oi, Kento Nakajima, Reo Matsumura, Tomoki Nakagawa, Takao Miyagawa, Kazuya Sakai, Ryo Saji, Hayato Taniguchi, Kohei Takahashi, Takeru Abe, Masayuki Iwashita, Mototsugu Nishii, Ichiro Takeuchi

**Affiliations:** Department of Emergency Medicine, Yokohama City University, School of Medicine, 3-9 Fukuura, Kanazawa-ku, Yokohama, Kanagawa 236-0004 Japan

**Keywords:** COVID-19, Syndecan-1, Endothelial injury, Glycocalyx, Severity, Classification

## Abstract

**Background:**

Coronavirus disease 2019 (COVID-19) pneumonitis associated with severe respiratory failure is associated with high mortality. The pathogenesis of COVID-19 is associated with microembolism or microvascular endothelial injuries. Here, we report that syndecan-1 (SDC-1), a component of the endothelial glycocalyx, may be a biomarker of severity classification for COVID-19 related to endothelial injury.

**Methods and analysis:**

We analyzed the data of COVID-19 patients for 1 year from February 2020 at Yokohama City University Hospital and Yokohama City University Medical Center Hospital. We selected COVID-19 patients who required admission care, including intensive care, and analyzed the classification of severe and critical COVID-19 retrospectively, using various clinical data and laboratory data with SDC-1 by ELISA.

**Results:**

We analyzed clinical and laboratory data with SDC-1 in five severe COVID-19 and ten critical COVID-19 patients. In the two groups, their backgrounds were almost the same. In laboratory data, the LDH, CHE, and CRP levels showed significant differences in each group (*P* = 0.032, *P* < 0.0001, and *P* = 0.007, respectively) with no significant differences in coagulation-related factors (platelet, PT-INR, d-dimer, ISTH score; *P* = 0.200, 0.277, 0.655, and 0.36, respectively). For the clinical data, the SOFA score was significantly different from admission day to day 14 of admission (*p* < 0.0001). The SDC-1 levels of critical COVID-19 patients were significantly higher on admission day and all-time course compared with the levels of severe COVID-19 patients (*P* = 0.009 and *P* < 0.0001, respectively).

**Conclusions:**

Temporal change of SDC-1 levels closely reflect the severity of COVID-19, therefore, SDC-1 may be a therapeutic target and a biomarker for the severity classification of Covid-19.

## Introduction

The novel coronavirus disease 2019 (COVID-19) pandemic, as declared by the World Health Organization, is caused by the severe acute respiratory syndrome coronavirus 2 (SARS-CoV2) [1, 2] with an overall mortality rate of approximately 31% of those admitted to an Intensive Care Unit (ICU) [3]. Recent studies have reported a high prevalence of thrombotic events in COVID-19, similar to the high venous thromboembolism (VTE) rates observed in patients with other viral pneumonias, including severe acute respiratory syndrome (SARS) and Middle East respiratory syndrome (MERS-CoV) [4–7]. Based on the changes in the plasma levels of d-dimers, fibrinogen-degradation products, and antithrombin, a prothrombotic state in COVID-19 has been suggested [6]. Furthermore, an elevated d-dimer level at hospital admission has also been associated with increased odds of in-hospital death [8]. Thrombotic complications and micro-thrombosis in the pulmonary vasculature have also been observed on autopsies on COVID-19 patients [9, 10]. While the mechanisms underlying thrombotic risk in COVID-19 patients are unclear, thrombosis developed in critically ill patients through the dysregulation of coagulation and/or endothelial injury [11, 12]. Therefore, venous abnormalities have emerged as important considerations in the management of hospitalized patients with COVID-19.

Microvascular endothelial cell injury also precipitates thrombosis [11], with or without coagulation abnormalities, particularly in the alveolar capillary where COVID-19 pneumonia and lung injury are observed clinically [10]. Finally, platelet adhesion to the microvasculature is largely inhibited by the glycocalyx, a gel-like substance that coats the luminal surface of endothelial cells [13, 14]. The glycocalyx modulates leukocyte–endothelial interactions, thrombus formation, and other processes that lead to microcirculatory dysfunction and critical organ injury in sepsis. It also acts as a regulator of vascular permeability and contains mechano-sensors as well as receptors for growth factors and anticoagulants. Inflammation-induced degradation of the glycocalyx is thought to contribute to microvascular pathology and thrombosis formation in sepsis of various etiologies. In a component of the endothelial glycocalyx, we focused on syndecan-1 (SDC-1) because the role of SDC-1 can be induced on the surface of endothelial cells in the context of wound healing and inflammation, suggesting a role for syndecan-1 in the interaction of leukocytes with endothelial cells,

The overall aim of this hypothesis-generating study was to characterize the changes of SDC-1, during the hospitalization of COVID-19 patients and it usefulness as a biomarker for the severity classification of COVID-19 in terms of endothelial injury.

### Patients

In this retrospective study, we used the data of COVID-19 patients (except for those without symptoms or mild symptoms) who underwent standard treatment, including intensive care at our department of the Yokohama City University Hospital and Yokohama City Hospital Center Hospital from February 2020 to December 2020, to analyze their clinical and biological features. Risk factors, morbidity, and mortality outcomes were also analyzed. Comorbidities were obtained for each patient, and outcome data were obtained at the follow-up in our hospital.

### Ethical approval

This study for all clinical data, material and therapies was approved by the Ethics Committee of the Yokohama City University School of Medicine (approval number B200200047). Consent for participation in this study was obtained by a description of how to opt out. (https://www.yokohama- cu.ac.jp/amedrc/ethics/ethical/fuzoku_optout.html). Written informed consent was obtained from each patient or family before treatment.

## Methods

Disease severity was categorized into four stages, that is, mild, moderate, severe, and critical, based on previously published guidelines on the diagnosis and treatment of novel coronavirus [15]. Briefly, mild cases were defined as no symptoms and no need of oxygen without pneumonia in computed tomography (CT) scan. Moderate cases were defined as mild respiratory symptoms, radiological evidence of pneumonia, and 93% < SpO_2_ < 96%. Severe cases were defined as SpO_2_ ≤ 94%, requiring oxygen support. Critical was defined as requiring heart–lung machine or extracorporeal membrane oxygenation (ECMO) support for the acute respiratory distress syndrome (ARDS). We combined this with severe and critical, referred to as critical in our study.

We collected blood samples daily, starting at admission depending on the clinical condition of the severe COVID-19 patients who needed oxygen and the critical ones who needed intubation management. The severity classification of treatment strategies for the patients with critical COVID-19 are shown in Table 1. Upon admission to our hospital, we made a diagnosis of COVID-19 using a positive reverse transcriptase–polymerase chain reaction (RT-PCR) assay for SARS-CoV-2 in the respiratory tract and laryngeal swab samples tested by a designated diagnostic laboratory.

**Table 1:** Therapeutic Strategy for critical COVID-19

● Therapeutic strategy for critical COVID-19 [16]		
1) Mechanical ventilator (primary setting)	mode	pressure control
	PEEP	10-15 cmH_2_O
	Driving Pressure	6-8/BW (kg) cmH_2_O
	Respiratory Rate	12-16/min.
2) Antiviral therapy	Loponavir/Ritonavir, Fapiviravir, Remdesivir [17]	10 days
3) Systemic steroid therapy	Dexamethasone [18]	10 days
4) Anticoagulant therapy	UFH with therapeutic dose according to APTT (1.5-2 times as normal)	
5) Protection for DVT	Intermittent air compression and elastic stocking	
6) Antibiotics	for CAP or secondary bacterial or fungus infection	
7) Rehabilitation	early intervention by NS, PT and OT	
8) Nutrition	early intervention via tube feeding or TPN	
9) Supportive therapy	sedation,catecholamine support etc. via central venous catheter Extracorporeal membrane oxygenation (ECMO)	

*BW* Body Weight, *PEEP* positive end-expiratory pressure, *UFH* unfractionated heparin, *APTT* activated partial thromboplastin time, *CAP* community associated pneumonia, *NS* nurse, *PT* physical therapist, *OT* occupational therapist, *TPN* total parenteral nutrition

We also recorded the clinical interventions made during the observation period, including the use of antibiotics, antiviral agents, systemic corticosteroids, vasoactive medications, venous thromboembolism prophylaxis, antiplatelet or anticoagulation treatment, renal replacement therapy, high-flow oxygen therapy, and mechanical ventilation (invasive and noninvasive). Once the first five severe COVID-19 patients were enrolled, 10 patients with critical COVID-19 were matched by characteristics.

### Data collection

Patients were followed up until hospital discharge or death. The patient information that was collected included demographic characteristics, pre-existing comorbidities, Acute Physiology and Chronic Health Disease Classification System II (APACHE II) scores on the date of hospitalization, and laboratory tests.

The laboratory tests included several hemostatic parameters, such as white blood cell counts (WBC), hemoglobin levels, platelet (Plt) counts (in complete cell count), prothrombin times, international normalized times (PT-INR), d-dimer levels (in plasma), lactic acid dehydrogenase (LDH) and organ parameters, such as liver function enzymes (transaminase [AST, ALT], cholinesterase [CHE]), pancreatic enzymes (amylase and lipase), renal function tests (BUN, Cr), and electrolytes (in serum) were measured in each group at each time point (day 1, 2, 3, 5, 7, 10, and 14) from day 1 to day 14 after admission as general laboratory test.

The severity of illness was evaluated according to the Sequential Organ Failure Assessment (SOFA) and APACHE II scores. The APACHE II score was evaluated on day 1, and the SOFA score was evaluated at each time point from day 1 to day 14. The incidence of disseminated intravascular coagulation (DIC) was evaluated at each time point during hospitalization based on the International Society on Thrombosis and Hemostasis (ISTH) overt DIC and the Japanese Association for Acute Medicine (JAAM) DIC criteria. We used prophylactic unfractionated heparin (UFH) for both the COVID-19 and control groups when patients had a high risk of VTE, such as severe obesity, cancer, orthopedic surgeries, and prior histories of VTE.

### Sample collection

Blood Draws Standard operating procedures were used to ensure that all the samples were treated rapidly and equally. Blood was obtained from critically ill ICU patients via indwelling catheters daily in the morning and placed to the laboratory as a routine laboratory test. About ELISA sample, a whole blood sample was placed immediately on ice, and once transferred to a negative pressure hood, blood was centrifuged and plasma was isolated, aliquoted at 500 μL, and frozen at − 80 °C. All the samples remained frozen until use, and the freeze/thaw cycles were minimized. All the plasma analytes were measured in duplicate with enzyme-linked immunosorbent assays (ELISA) as per the manufacturer’s recommendation. Analytes measured SDC-1 (CD138, Diaclone Cat. No: 950.640.096).

### Statistical analysis

Patients were divided into two groups for comparison: severe and critical groups. For each group, medians (interquartile ranges) and frequencies (%) were reported for continuous and categorical variables, respectively. We used the Mann-Whitney U test for continuous variables and Fisher’s exact test for the categorical variables, using patient characteristics, standard blood tests, physical condition, and SDC-1 levels. In addition, repeated measures analyses of variance (ANOVA) were used to evaluate group and time differences, as well as their interactions, for Plt, PT-INR, d-dimer, WBC, CRP, LDH, CHE, SOFA score, ISTH score, and SDC-1. Two-sided *P* values of less than 0.05, were considered statistically significant. All the population statistics were conducted using JMP Pro Version 15 (SAS Institute Inc., Cary, NC, USA) and IBM SPSS Statistics for Windows, Version 25.0 (Armonk, NY: IBM Corp).

## Results

We investigated five patients with severe symptoms and 10 patients with critical symptoms with a positive diagnosis of COVID-19 (median age, 73.0 [IQR, 67–77] years). The critical group consisted with three patients managed with mechanical ventilation, four ECMO patients and three death patients. Serum and plasma samples that could be tested retrospectively were extracted from the admission day to day14 after admission because all the clinical and test data were collected at an accurate time point. Baseline demographic characteristics, admission day after onset, comorbidities, and APACHE II score on admission day are reported in Table 2. All patients had typical COVID-19 pneumonia findings on CT scans, such as bilateral grand glass opacities (GGOs), and consolidations caused respiratory insufficiency with associated hypoxemia in both groups. The day of admission after onset was significantly different in both groups (*P* = 0.0160). All the other reported baseline measures were nonsignificant between the patients.

**Table 2:** Patients’ Characteristics

Patient	Severe (*n* = 5)	Critical (*n* = 10)	*p*-value
Age (year-old; median (IQR))	73 (57-75)	73 (67-78)	0.8537
Gender (Male: %)	40%	70%	0.3286
BMI (median (IQR))	22.8 (22-24)	25.6 (23-28)	0.4990
First Symptom (%)			
dyspnea	40	20	0.5604
fever	75	80	0.5604
cough	20	20	1.0000
others	40	20	0.5604
Smoking History (%)	75	40	0.6084
Admission day after onset (day; median (IQR))	3 (2-3)	7 (6-8)	**0.0160**
Complications (%)			
Diabetes	40	60	0.6084
Renal Dysfunction	20	10	1.0000
Hemodialysis	20	10	1.0000
Hypertension	40	40	1.0000
Hyperlipidemia	0	10	1.0000
Hyperuricemia	0	20	0.5238
Cardiovascular Disease	20	0	0.6084
Respiratory Disease	75	20	0.2507
Cancer	20	10	1.0000
Collagen Disease	20	0	0.3333
Thrombotic disease	0	0	1.0000
others	2	0	0.0952
APACHE II Score (pts; median (IQR)	5 (5-8)	5 (5-10)	0.2313

*Mann-Whitney U test or Fisher’s exact test; *IQR* interquartile range, *BMI* Body Mass Index

**Fig. 1:**
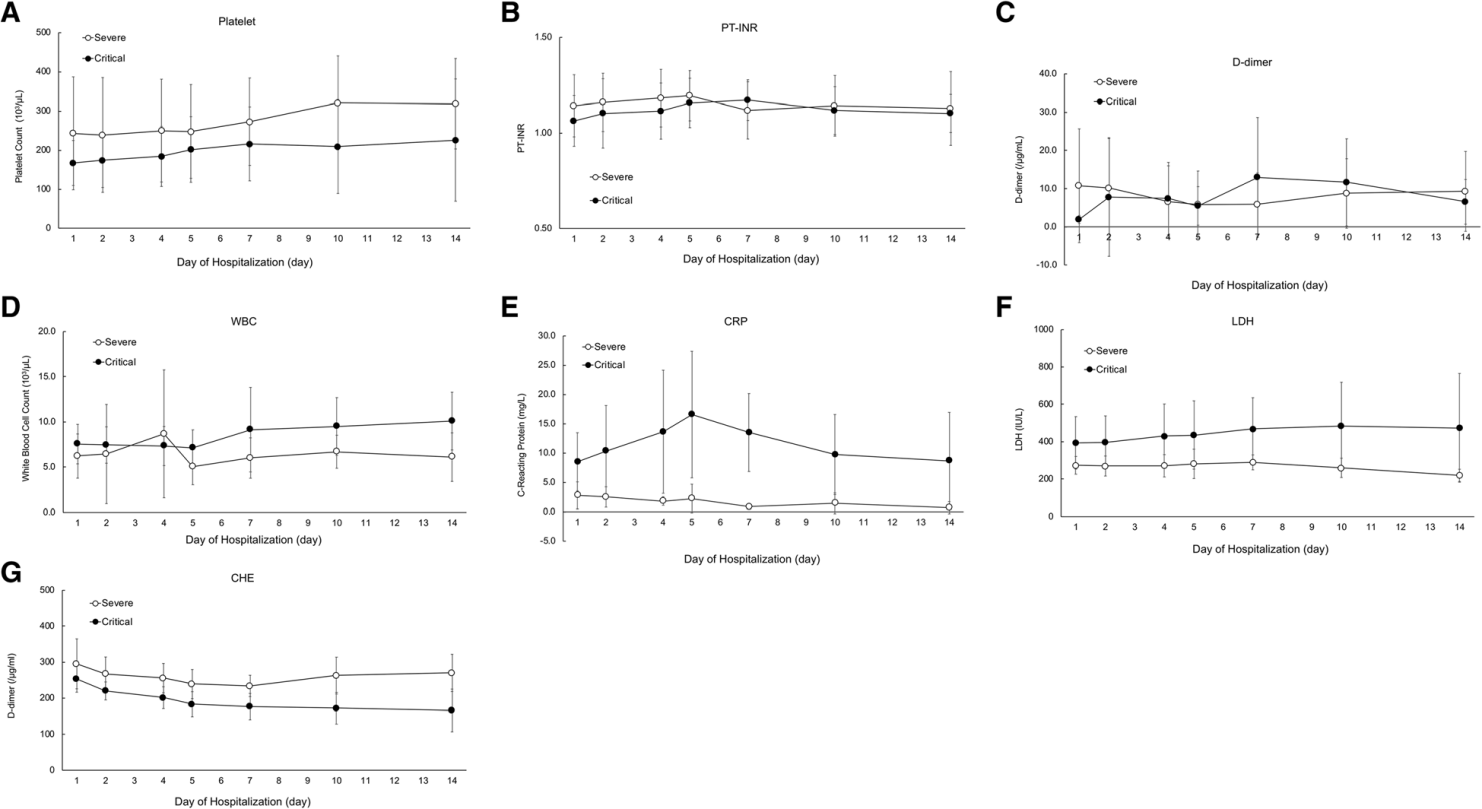
Time course for each laboratory data between severe and critical COVID-19 patients from admission day to day14 of hospitalization from admission day. (**A**) platelet, (**B**) PT-INR, (**C**) d-dimer, (**D**) WBC, (**E**) CRP, (**F**) LDH and (**G**) CHE. Daily values are represented as means (± sem; *P < 0.05)

Regarding hemostatic parameters, platelet counts over time during the observational period were not significantly different between the two groups (Ps: 0.200, 0.056, 0.902, for group, time differences, and their interaction, respectively) (Fig. 1A), and the severe group had consistently higher platelet counts from the beginning of hospitalization than the critical group. Similarly, there were no significant differences in PT-INR (Ps: 0.227, 0.686, 0746, for group, time differences, and their interaction, respectively) and d-dimer levels (Ps: 0.655, 0.695, 0.220, for group, time differences, and their interaction, respectively) between the two groups (Fig. 1B, C). Therefore, the hemostatic parameters were not significantly different between the two groups in our study.

In the parameters of each organ, WBC had no significant difference between the two groups (Ps: 0.219, 0.299, 0.163, for group, time differences, and their interaction, respectively) (Fig. 1D), but the C-reactive protein (CRP), reported as a prognostic factor, was significantly different between the two groups (Ps: 0.007, 0.257, 0.257, for group, time differences, and their interaction, respectively) (Fig. 1E). Regarding organ biomarkers, there were almost no significant differences between the two groups. However, the LDH (Ps: 0.032, 0.949, 0.784, for group, time differences, and their interaction, respectively) and CHE (Ps: < 0.0001, 0.002, 0.138, for group, time differences, and their interaction, respectively) levels were significantly different between the two groups (Fig. 1F, G).

**Fig. 2:**
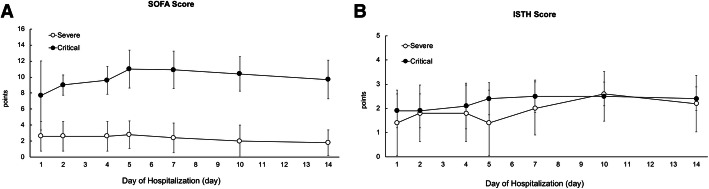
Time course for SOFA score (**A**) and ISTH score (**B**) between severe and critical COVID-19 patients from admission day to day14 of hospitalization from admission day. Daily values are represented as means (± sem; *P < 0.05)

In terms of severity of illness and the coagulation scores, the SOFA scores were significantly different between the two groups (Ps: < 0.0001, 0.236, 0.163, for group, time differences, and their interaction, respectively) (Fig. 2A) because critical patients needed to reduce their levels of consciousness with sedation to enable intensive care with mechanical ventilation for respiratory disorders and; moreover, use vasopressors for the hypotension caused by sedation as a counter-measure, compared to severe patients. In contrast, the ISTH scores were not significantly different between the two groups (Ps: 0.360, 0.041, 0.608, for group, time differences, and their interaction, respectively) (Fig. 2B).

**Fig. 3:**
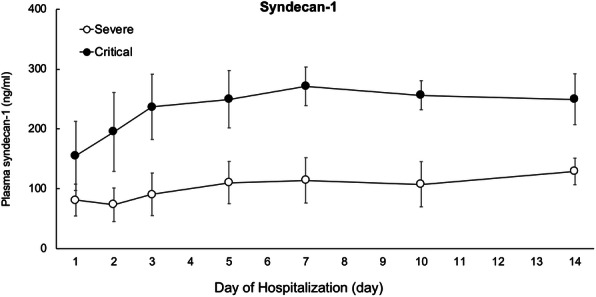
Time course for syndecan-1 (SDC-1) between severe and critical COVID-19 patients. SDC-1 remained elevated until day14 of hospitalization from admission day. Daily values are represented as means (± sem; *P < 0.05)

We measured SDC-1, one of the components of the glycocalyx as an endothelial cell injury marker in plasma using ELISA. Figure 3 shows that the SDC-1 levels were elevated significantly in the critical COVID-19 patients compared with the severe COVID-19 patients, with significant differences on admission day and over time in both groups (Ps: < 0.0001, < 0.0001, 0.009, for group, time differences, and their interaction, respectively). The data suggests that SDC-1 levels reflect the severity of disease and the main pathology of vasculitis in the lungs.

## Discussion

In this study, we analyzed the relationship between the severity of COVID-19 and SDC-1, focusing on one of the components of the glycocalyx as an indicator of endothelial injury with coagulopathy and as a biomarker for the severity of COVID-19. Previous reports that have been extensively cited [16, 17], have suggested that the hyperactivation of the inflammatory cascade, leading to cytokine storm, is a critical biological response in patients with severe COVID-19. From regular blood tests, d-dimer, LDH, CHE, and CRP levels have been reported previously as potential parameters for severity [16, 17]. In our study, although LDH, CHE, and CRP levels were significantly different between patients with severe and critical COVID-19 indicating their potential as parameters for severity, the d-dimer levels were not significantly different. Significantly elevated levels of the inflammatory cytokines TNF-α, IL-1, IL-6, IFN-λ3, IL-6, IP-10, and CXCL9 have been documented in severe COVID-19 compared to non-severe disease cases [18–22]. In our study, we did not focus on inflammatory cytokines for the COVID-19 classification of severity.

It has been reported that COVID-19 respiratory distress may be due to micro-emboli or endothelial injuries associated with respiratory deterioration and death [8, 23]. In addition, many critical COVID-19 patients developed VTE, which appeared to be related to coagulopathy [6, 24]. In particular, VTE emerged as an important consideration in the management of hospitalized patients with COVID-19. In recent years, common pathways for venous thrombosis have been described, with inflammation and hypercoagulation being key factors in the mechanism of venous thrombotic events [25]. These concerns should be balanced by emerging data that the incidence of VTE in hospitalized critical COVID-19 patients or in ICU settings were higher than that reported by historical data in similar patients, with an incidence of VTE of 27% in a previous study using standard thromboprophylaxis and an incidence of 25% in another study without prophylaxis [4, 6, 26]. Although the mechanisms underlying vascular thrombosis in COVID-19 have not yet been clearly defined, several have been postulated. Activation of the complement system leads to endothelial cell injury and death with subsequent vascular denudation and exposure of the thrombogenic basement membrane, which drives the activation of clotting cascades. These events result in inflammation, microvascular thrombosis, vessel edema, and hemorrhagic sequelae, all of which are prominent features of lung pathology in patients with COVID-19-associated pneumonia [27]. In an autopsy study of ten patients with COVID-19, small vessel thrombi formation in the lung peripheries were associated with foci of alveolar hemorrhages [28]. Many researchers have focused on coagulopathy in COVID-19 by investigating coagulation-related factors. In our study, the coagulation factors platelet count, PT-INR level, and d-dimer level were not significantly different between the two groups. In preliminary data (data no shown), there was significant difference on d-dimer levels between mild-moderate and severe-critical COVID-19. But, there was no significant difference on d-dimer levels between severe and critical COVID-19 patients. As one of the possibility, we think that the severe or critical COVID-19 patients that require oxygen therapy may be strongly induced microvasculitis and microthrombosis in the lungs (mild-moderate patients had less microvasculitis and microthrombosis in the lungs, so these patients probably needed no oxygen supply), thus maybe there was no significant difference between severe and critical COVID-19 patients. In addition, the ISTH score, as an index for evaluating coagulopathy, also showed no significant differences. Since we administered therapeutic doses of anticoagulant therapy targeting the micro-thromboembolism of COVID-19 in all the cases, many coagulant factors other than platelet count may have been improved by anticoagulant therapy. Therefore, it may not be useful as a predictor of the severity of COVID-19. For anticoagulant therapy, while the World Health Organization (WHO) recommended therapeutic anticoagulation rather than intermediate dosing [29] the optimal thrombo-prophylactic strategy in the critically ill hospitalized COVID-19 patient population remains uncertain (conditional recommendation, very low certainty). However, the thrombotic tendencies in COVID-19 promote VTE formation in intensive care management, so we suggest that therapeutic anticoagulant doses are more appropriate than intermediate-dose anticoagulants.

In contrast, we focused on endothelial injury by SDC-1 as a part of the glycocalyx. Our data indicate increased SDC-1 and continuous changes in SDC-1, a core protein of the glycocalyx whose degradation indicates endothelial injury [30–32] in critical COVID-19 patients relative to severe COVID-19 patients at 2 weeks of admission. SDC-1 level at 2 weeks of admission showed no significant differences in critical COVID-19 patients, under mechanical ventilation, ECMO management, and death. SDC-1 levels in critical COVID-19 patients have been reported to be related to respiratory disorders of COVID-19 [33–35]. These reports showed that critically ill patients with COVID-19 had higher SDC-1 levels than healthy controls. Therefore, the relationship between treatment progress and SDC-1 levels in critically ill patients with COVID-19 remains controversial. Our data suggest that COVID-19 results in endothelial injury and the degradation of the endothelial glycocalyx. The SDC-1 levels were all elevated significantly on admission day in the plasma of COVID-19 patients and they remained elevated persistently, in the plasma up to day 14 after admission. Oda et al. reported that 78 healthy individuals receiving no treatment and with no relevant medical history or laboratory data reported a median SDC-1 concentration of 19.3 [36]. As a preliminary experiment (data not shown), we analyzed the SDC-1 levels of COVID-19 negative pneumonia patients suspected of COVID-19 pneumonia when they presented at our department with some symptoms. Compared with a volunteer healthy control, there were significant differences in the SDC-1 levels between the COVID-19 positive patients and the healthy controls (SDC-1 concentration of healthy control 23.6 ng/ml; *P* = 0.043), nevertheless there were no significant differences with the COVID-19 negative pneumonia group (SDC-1 concentration of COVID-19 negative pneumonia patient 40.8 ng/ml; *P* = 0.238). The glycocalyx is a complex structure composed of glycosaminoglycans (e.g., hyaluronic acid and chondroitin sulfate), proteoglycans (e.g., syndecan-1 and heparan sulfate), and various plasma proteins (e.g., albumin and antithrombin). Disturbance of the glycocalyx, often due to the increased expression and release of proteinases and glycosidases (e.g., hyaluronidases, sheddases, and matrix metalloproteinases), and has profound consequences on vascular function [37]. For example, loss of glycocalyx components decreases nitric oxide production and increases oxidant production, thereby facilitating ligand-receptor interactions and subsequent platelet recruitment to the vascular endothelium [38]. SDC-1 is a proteoglycan containing both heparan- and chondroitin-sulfate chains that mediate cellular responses to signaling molecules as well as cell-cell and cell-matrix interactions [39]. During inflammation, the SDC-1 inhibits neutrophil adhesion and migration. The shedding of SDC-1 from the cell surface is initiated by the heparanase-dependent removal of the heparan-sulfate side chains [40], thereby instigating subsequent cleavage of the core SDC-1 protein by enzymes such as matrix metalloproteinases. Moreover, while moderate SDC-1 shedding is thought to aid in resolving inflammation, excessive shedding is likely pathogenic, as the complete loss of SDC-1 allows for increased leukocyte adhesion and recruitment across the endothelial monolayer, as well as enhanced platelet aggregation and adhesion. Platelet interaction with activated pulmonary endothelial cells, is at least in part due to the glycocalyx degradation and the subsequent decrease in nitric oxide production which promotes vascular occlusion, enhances inflammation, and drives viral pathogenesis. Inhibition of this interaction, through antiplatelet or thrombolytic therapies, can represent a potential therapeutic strategy (i.e., using reduced doses of recombinant tissue-type plasminogen activator over prolonged periods) for the treatment of severe viral infections, such as COVID-19. Therefore, our analysis indicated that SDC-1 may be a biomarker for the severity of severe COVID-19. Similar to our results, recently published article reported SDC-1 levels alone or combined with other markers like cytokines might be a good candidate for disease activity monitoring [41]. In addition, if some treatment to remove glycocalyx from the endothelium can be established, it may contribute to a reduced oxygen administration period, and shortened mechanical ventilation and ECMO administration periods, thereby improving the prognosis, and reducing unexplained sequelae after COVID-19, including respiratory symptoms.

Our study identified a unique prothrombotic state in critically ill COVID-19 patients that may be amenable to therapeutic targeting. However, our study had several limitations. First, this study was performed in a single hospital with a small study population, since there are currently few confirmed and recovered cases of COVID-19 in Japan. Therefore there may have been selection bias, in that the clinical data of our selected cases were known and while their serum was collected according to the protocol from the day of admission to the 14th day of admission without intentional selection, we should have analyzed all patients in our institute according to strict protocol. Second, no therapeutic treatment for VTE in critical COVID-19 patients was available for use in a parallel control group. However, we believe that the credibility of the therapeutic effect is high, as our study provided a comprehensive examination, including clinical features, laboratory findings, and physical findings, in a single institution. These hypothesis-generating results are valuable for future studies on antithrombotic therapies and clinical trials. Finally, we reported mortality as a clinical outcome in our COVID-19 patients; however, future studies with larger sample sizes can explore whether reported changes in thrombotic factors and endothelial injury markers correlate with additional clinical outcomes such as hospital stay or mortality. The beneficial effects of specific therapeutic strategies may be diluted by patient and disease heterogeneity, suggesting that a personalized treatment approach is required. Our study revealed significantly elevated SDC-1 levels in COVID-19 patients, suggesting that therapies to coagulopathy and to protect/restore the glycocalyx may be therapeutically indicated. One possibility is that recombinant thrombomodulin (rTM), which is used to treat DIC, may be effective as a therapeutic agent because rTM treatment affects inflammation, cell proliferation/differentiation, and glycocalyx synthesis in serum and lung tissue, subsequently attenuating the ARDS caused by endothelial injury in animal experimental models. Further clinical studies are required to validate this concept.

## Conclusion

We suggest that it is important to analyze the temporal change of SDC-1 levels of COVID-19 patients to determine their severity and prognosis. Further studies on detailed and early laboratory, clinical, and imaging characterizations are required to better understand the pathophysiology of endothelial injury in COVID-19 patients. Thus, the treatment focused on the degree of vasculitis, including glycocalyx, may be one of the treatment options for COVID-19.

## Data Availability

Data requests should be made to the corresponding authors.

## Abbreviations

COVID-19: Coronavirus disease;
SARS-CoV-2: Severe acute respiratory syndrome coronavirus 2
ICU: Intensive Care Unit
MERS-CoV: Middle East respiratory syndrome
VTE: Venous thromboembolism
SDC-1: Syndecan-1
CT: Computed tomography
ECMO: Extracorporeal membrane oxygenation
ARDS: Acute respiratory distress syndrome
RT-PCR: Reverse transcriptase–polymerase chain reaction
APACHE II: Acute Physiology and Chronic Health Disease Classification System II
WBC: White blood cell
Plt: Platelet
PT-INR: Prothrombin international normalized times
LDH: Lactic acid dehydrogenase
CHE: Cholinesterase
SOFA: Sequential Organ Failure Assessment
DIC: Disseminated intravascular coagulation
ISTH: International Society on Thrombosis and Hemostasis
JAAM: Japanese Association for Acute Medicine
UFH: Unfractionated heparin
ELISA: Enzyme-Linked Immuno Sorbent Assay
ANOVA: Analyses of variance
CRP: C-reactive protein
GGOs: Grand glass opacities
WHO: World Health Organization
rTM: Recombinant thrombomodulin
